# *Clin.iobio*: A Collaborative Diagnostic Workflow to Enable Team-Based Precision Genomics

**DOI:** 10.3390/jpm12010073

**Published:** 2022-01-08

**Authors:** Alistair Ward, Matt Velinder, Tonya Di Sera, Aditya Ekawade, Sabrina Malone Jenkins, Barry Moore, Rong Mao, Pinar Bayrak-Toydemir, Gabor Marth

**Affiliations:** 1 Department of Human Genetics, University of Utah School of Medicine, Salt Lake City, UT 84112, USA; matt.velinder@utah.edu (M.V.); tony.disera@gmail.com (T.D.S.); aditya.ekawade@gmail.com (A.E.); barry.moore@genetics.utah.edu (B.M.); 2 Frameshift Labs, Inc., Cambridge, MA 02142, USA; 3 Department of Pediatrics, University of Utah Scbool of Medicine, Salt Lake City, UT 84112, USA; Sabrina.MaloneJenkins@hsc.utah.edu; 4 ARUP Laboratories, Department of Pathology, University of Utah School of Medicine, Salt Lake City, UT 84112, USA; rong.mao@aruplab.com (R.M.); pinar.bayrak-toydemir@aruplab.com (P.B.-T.)

**Keywords:** genomics, clinical, software, visualization, collaboration, diagnostics, genetics, rapid sequencing, NICU, undiagnosed disease, reanalysis

## Abstract

The primary goal of precision genomics is the identification of causative genetic variants in targeted or whole-genome sequencing data. The ultimate clinical hope is that these findings lead to an efficacious change in treatment for the patient. In current clinical practice, these findings are typically returned by expert analysts as static, text-based reports. Ideally, these reports summarize the quality of the data obtained, integrate known gene–phenotype associations, follow allele segregation and affected status within the sequenced samples, and weigh computational evidence of pathogenicity. These findings are used to prioritize the variant(s) most likely to cause the given patient’s phenotypes. In most diagnostic settings, a team of experts contribute to these reports, including bioinformaticians, clinicians, and genetic counselors, among others. However, these experts often do not have the necessary tools to review genomic findings, test genetic hypotheses, or query specific gene and variant information. Additionally, team members often rely on different tools and methods based on their given expertise, resulting in further difficulties in communicating and discussing genomic findings. Here, we present *clin.iobio*—a web-based solution to collaborative genomic analysis that enables diagnostic team members to focus on their area of expertise within the diagnostic process, while allowing them to easily review and contribute to all steps of the diagnostic process. *Clin.iobio* integrates tools from the popular *iobio* genomic visualization suite into a comprehensive diagnostic workflow, encompassing (1) genomic data quality review, (2) dynamic phenotype-driven gene prioritization, (3) variant prioritization using a comprehensive set of knowledge bases and annotations, (4) and an exportable findings summary. In conclusion, *clin.iobio* is a comprehensive solution to team-based precision genomics, the findings of which stand to inform genomic considerations in clinical practice.

## 1. Introduction

As genomic sequencing continues to decrease in cost, its use as a powerful, cost-effective clinical test has been established [1,2]. This is especially true for the diagnosis of suspected genetic conditions in rare diseases and critically ill newborn settings [3,4,5]. Bioinformatic pipelines to map a patient’s genomic data to a reference genome and determine high-confidence variant calls have become increasingly standardized, and can be deployed with relative ease in both academic and commercial settings. However, the path to diagnosis remains complex, and expert, team-based interpretation of potentially causative candidate variants remains a significant bottleneck. Reaching a diagnostic decision often requires the collaboration of bioinformatics analysis teams, genetic counselors, and clinical geneticists, spanning a diverse range of expertise. While each case is unique and workflows differ between clinical settings, the following steps are essentially always required: (1) assessment of data quality; (2) identification of candidate genes, based on relevant phenotype and disease terms; (3) interpretation of candidate diagnostic variants, within the context of both computationally prioritized and phenotype-prioritized genes; and (4) reporting variant findings to clinical teams for final diagnostic decisions. Based on these universal workflow steps, we have developed a web-based tool, *clin.iobio*, to support a team-based approach to genomic diagnostics, focusing on ease of use, accessibility, and collaboration.

Data quality assessment is an often overlooked but critical component of all genomic analyses, and is typically performed by bioinformaticians. In many clinical diagnostic settings, these quality metrics are buried within reports or omitted entirely. However, data deficiencies can dramatically affect downstream analyses. Important sequencing data quality metrics typically include the overall sequencing coverage/depth across target sequence regions (exome or genome) and the distribution of the types of variants called. Following data quality assessment, a differential clinical diagnosis approach, often performed by medical geneticists or genetic counselors, is typically employed to carefully review patient (and family member) phenotypes. These phenotype terms are then coded into standardized Human Phenotype Ontology (HPO) terms in order to generate a high-confidence list of phenotype-associated genes [6]. Variants within these genes have a higher likelihood of explaining the patient’s phenotype and, consequently, require thorough investigation. Depending on the clinical setting, patients may also present with a large number of non-specific phenotypes where the input of multiple team members can help to refine clinical diagnoses and prioritize the most objective and specific phenotypes [7]. Additionally, proband phenotypes may change over time, requiring revisions to patient phenotype terms; thus, it is necessary that phenotype terms and prioritized genes can be dynamically reviewed and updated.

Following phenotype description, variants are reviewed and interpreted. Typical trio sequencing results range from hundreds of thousands to millions of variants, in exome or genome studies, respectively. This number of variants can be significantly reduced by using computational methods that prioritize variants based on Mendelian modes of inheritance, population frequency, and predicted impacts on coding proteins [8,9,10,11]. However, these methods remain almost exclusively used by bioinformaticians, and require significant computational skills and resources; as such, clinical and genetics experts are typically only provided with static, text-based summaries of candidate variant information for their review. These summaries are often not sufficient to make a diagnostic decision about the variant, and this approach limits the ability of team members to contribute to diagnostic decisions. Lastly, written reports summarizing genomic findings typically require specific information from multiple team members based on their expertise, requiring additional communication exchanges. 

Many tools have attempted to address specific challenges within this typical clinical diagnostic workflow. However, these approaches have largely been developed in commercial settings, leaving few options for academic researchers. To date, no academic tools currently exist that provide a comprehensive, team-based, genomic diagnostic workflow. *Clin.iobio* was specifically designed as a solution to this challenging problem of team-based genomic diagnostics. We identified the major components of a typical genomic analysis workflow, and developed a framework that allows all team members to contribute their domain of expertise to diagnostic decisions via an intuitive web app that provides a comprehensive, dynamic, and collaborative workflow to potentially guide clinical practice based on genomic findings.

## 2. Results

The development of *clin.iobio* was guided by our collaboration with clinical teams in the rapid newborn intensive care unit (NICU) sequencing and undiagnosed disease clinics at the University of Utah. Rapid NICU sequencing programs rely on identifying diagnostic variants as quickly as possible, with the hope of impacting newborn clinical care as soon as possible. In these time-sensitive analyses, it is critical that all team members can review case information as soon as it is available. Here, we demonstrate the utility of *clin.iobio* using a representative case from our University of Utah rapid NICU sequencing program, where the clinical team achieved a rapid genetic diagnosis that informed clinical management.

*Clin.iobio* is routinely applied for all Utah NICU patient cases. These cases include many complex phenotypic presentations for which diagnostic decisions cannot be made by any available method, including *clin.iobio*, and remain undiagnosed. However, even in these undiagnosed cases, *clin.iobio* facilitates a team-based diagnostic process, allowing the team to confidently conclude that there is no feasible diagnosis at this time. Of the Utah NICU cases that were able to be diagnosed, *clin.iobio* prioritized the diagnostic variant(s) within minutes. Here, we describe a representative diagnostic case to highlight the *clin.iobio* analysis process. In this NICU case, a newborn was described to have a “fetal akinesia sequence” (HP:0001989) phenotype. Additional, less specific phenotypes included “arthrogryposis multiplex congenita” (HP:0002804), “elevated serum creatine kinase” (HP:0003236), “macrocephaly at birth” (HP:0004488), “nephrolithiasis” (HP:0000787), and “pulmonary hypoplasia” (HP:0002089). Our in-house variant alignment and calling pipeline provided *clin.iobio* with the necessary CRAM, VCF, and PED files for this case, and allowed our clinical diagnostic team to rapidly review the sequencing data and reach a diagnostic conclusion.

**Figure 1:**
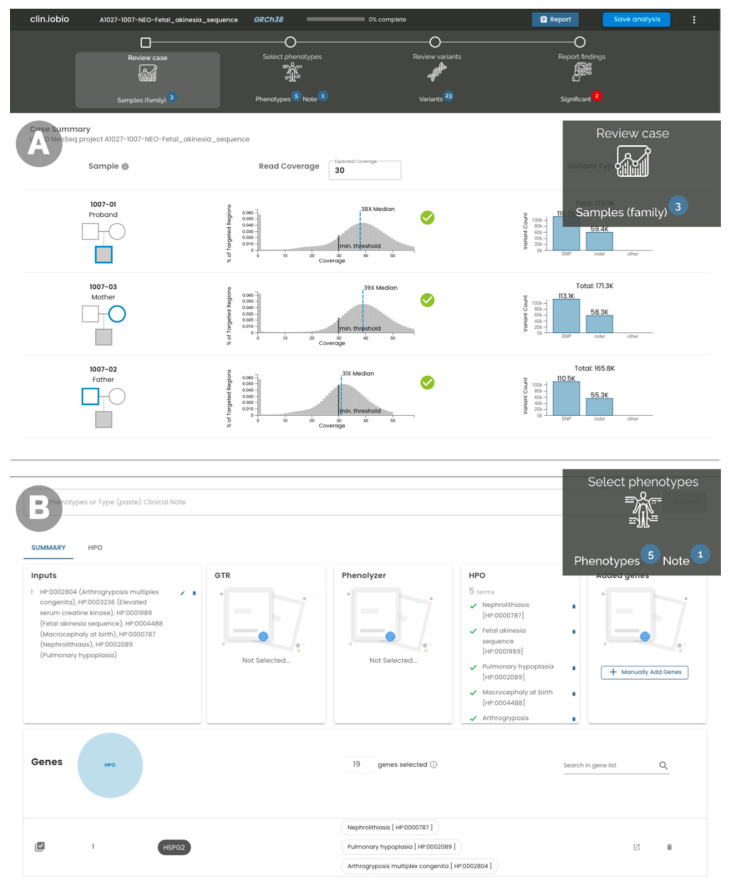
The first two steps in the *clin.iobio* web app, with the workflow shown at the top of the figure. This workflow is always present at the top of the page, with step-specific information (e.g., the number of identified significant variants) shown with each task. This workflow is not linear; rather, users can jump to whichever step they desire. (**A**) Basic overall quality control metrics for the patient and family members show that sequencing coverage has expected distributions, with median coverages above the required threshold. (**B**) A candidate gene list is generated and refined based on patient phenotypes. Here, a set of HPO terms was selected, and interactive charts limit the list to genes that are associated with at least 3 HPO terms.

**Figure 2:**
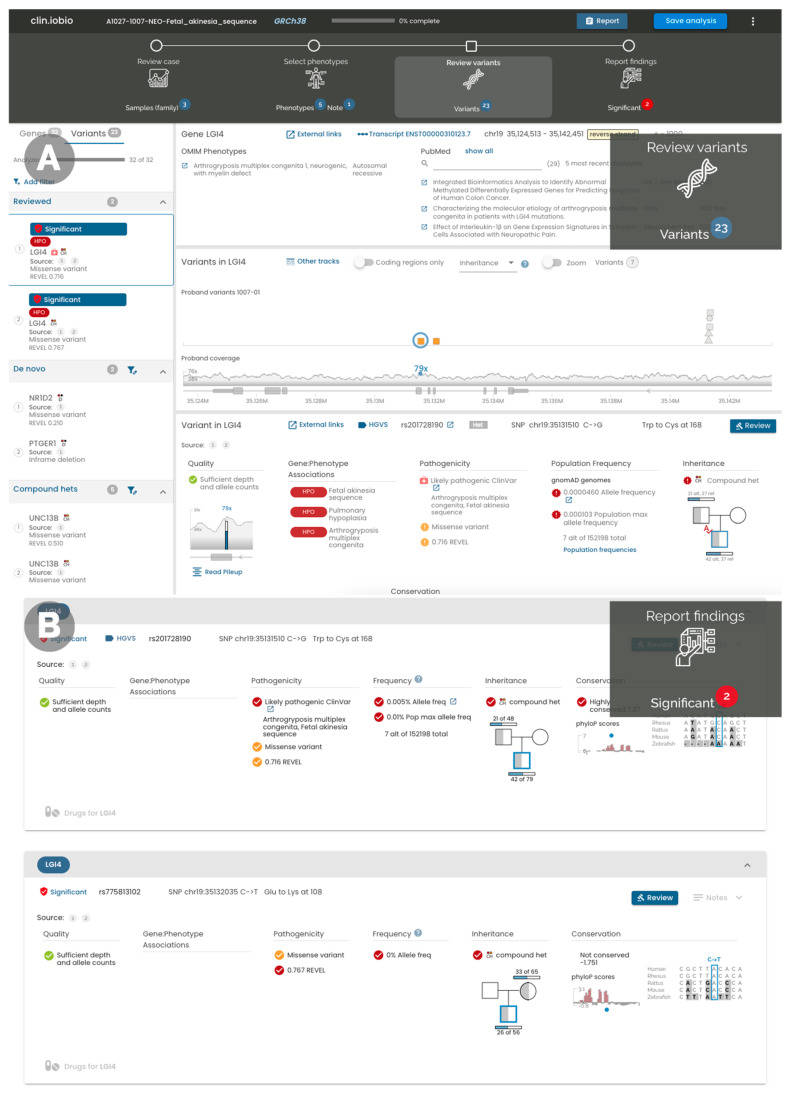
The final two steps in the *clin.iobio* web app, with the workflow shown at the top of the figure. (**A**) The variant review process includes all candidate variants in the left panel (variants that conform to a set of predefined filters). All variants in the selected *LGI4* gene are shown in the middle panel; one of the *LGI4* compound heterozygous variants is selected, showing variant-specific annotations in the bottom panel. This shows that the variant is listed as “likely pathogenic” in ClinVar, and is associated with relevant phenotypes; the gene–phenotype associations integrate information from the previous phenotype step. (**B**) The final step in the workflow summarizes information on the variants that have been marked as Significant or of Unknown Significance; this step acts as the starting point for a quick review of the case.

Our *iobio* suite comprises tools to perform specific focused analyses. For example, *gene.iobio* provides methods to interrogate individual variants in genes of interest. *Clin.iobio* differs from these existing tools, as it integrates the tools together into a comprehensive web-based diagnostic workflow, seamlessly passing information between steps and generating a final findings report. Each step in the *clin.iobio* workflow—and the tools used to power them—is described here for the representative NICU case. Stepwise within *clin.iobio*, the first step of data quality review revealed that all three individuals displayed the expected Poisson distribution of sequencing coverage, with median read coverages exceeding the minimum expected for this sequencing experiment (Figure 1A). In the second step of *clin.iobio*, clinically relevant HPO terms were entered into a freeform text box, where *clin.iobio* automatically interpreted the HPO syntax and generated a list of phenotype-associated genes (Figure 1B). In the initial analysis, the number of phenotype-associated genes was filtered to include only genes associated with three or more of the six provided HPO terms. As these genes were associated with multiple phenotypes present in the patient, they represented the genes most likely to explain the patient’s specific disease presentation. This dynamic filtering within *clin.iobio* resulted in 19 genes, limiting the number of variants for initial review to a manageable number. The dynamic design of *clin.iobio* allows this gene list to be expanded if the initial review results in no plausible candidate variants. The next step within *clin.iobio* is to review candidate variants (Figure 2A); this includes the variants in the 19 phenotypically prioritized genes, as well as candidate variants identified using upstream variant prioritization tools, e.g., Slivar [8] (see Materials and Methods). This integration with variant prioritization tools is critical in order to ensure that *clin.iobio* supports both phenotype- and gene-based approaches, as well as variants in genes not previously associated with the patient’s phenotypes. This variant review step is powered by our previously published *gene.iobio* [12] tool, whereby variants in all provided genes are annotated with a comprehensive set of annotations, including ClinVar [13], gnomAD [14], and REVEL [15] for missense variants. This variant prioritization step also provides OMIM-associated [16] genetic disorders and PubMed publications associated with the selected gene. Importantly, this *clin.iobio* workflow step also provides patient-specific gene–phenotype associations, as provided in the previous workflow step. Integrating this information in a single view enables team members to use primary sources and always up-to-date information in their interpretation of genetic variants. Within seconds, the *Review variants* step of *clin.iobio* annotated all variants within the provided gene list, prioritizing and prominently highlighting compound heterozygous variants in the *LGI4* gene. These were two missense variants (REVEL scores 0.716 and 0.767), where one variant was annotated as “likely pathogenic” in ClinVar and was associated with “arthrogryposis multiplex congenita” and “fetal akinesia sequence”—the most objective phenotypes provided for our patient. The sequence coverage of each variant was shown for all members of the pedigree, establishing that both variants were high-quality heterozygous variants and were present in the proband and one parent. Specifically for the missense variant in Figure 2A, the number of observations of the reference and alternate alleles was 42 and 37, respectively; the father had 21 and 27 observations, while the mother had 0 and 43, providing ample confidence in the called genotypes. The IGV [17] browser is integrated into this variant review step of *clin.iobio* to allow further read-level review if desired. Within *clin.iobio*, these variants were marked as significant, automatically populating the final report with this potentially diagnostic information (Figure 2B).

This representative case demonstrates how *clin.iobio* supports diagnosis in whole-genome sequencing data. The diagnostic workflow is largely the same when using whole-exome sequencing data, with the addition of extra quality control checks to account for the variable coverage in exome data. The minimum, median, and mean coverage in each exon are determined, and active warnings are provided for exons that fall outside of predefined and customizable thresholds. In exome data, the diagnostic team performs the same variant-level quality control checks as with genome data, but additionally can ensure that all exons in genes of interest are sufficiently covered, allowing team members to potentially identify false negatives due to low or absent coverage.

In this representative case, *clin.iobio* enabled a collaborative diagnostic approach that identified compound heterozygous *LGI4* variants. The patient’s clinical presentation began prenatally with polyhydramnios, arthrogryposis, and limited movement of fetal extremities. Pathogenic *LGI4* variants are associated with autosomal recessive arthrogryposis multiplex congenita, which the diagnostic team reviewed in conjunction with all other available information, including literature sources provided by *clin.iobio*, in the context of the patient. After comprehensive review, the team determined that these compound heterozygous *LGI4* variants were likely pathogenic, and were sufficient to return a genetic diagnosis to the family. Importantly, these genomic findings informed clinical care, including sparing the proband from additional invasive procedures and moving the patient towards palliative treatment. Additionally, this crucial prognostic and genetic information empowered the parents’ future family planning decisions. This real-world example demonstrates how *clin.iobio* provides an accessible team-based interface to enable the rapid identification of causative genetic variants and potentially inform clinical management.

## 3. Discussion

As genomic testing increasingly becomes a first-line diagnostic technique in many clinical settings, analysis and interpretation of genomic testing results demand significant effort from a multidisciplinary team of experts [18]. This team-based approach guarantees that the genomic data are comprehensively reviewed and all findings are discussed by bioinformaticians, geneticists, and clinical experts. However, this is a high-effort and low-throughput process, where all experts in the team are already extremely time-limited. As such, tools that enable team-based genomics but do not increase the workload of individual team members are urgently needed. We expect that this challenge will become increasingly apparent as the number of patients undergoing genetic testing continues to increase.

*Clin.iobio* was specifically designed to address the growing need to implement team-based genomic medicine. *Clin.iobio* is best suited for the analysis of monogenic Mendelian diseases, while providing a method for diagnostic teams to investigate all hypotheses in patient cases. Complex disorders—for example, those with multiple causative variants in multiple genes, especially when these include variants of unknown significance—remain challenging to interpret using any genomic diagnostic tool, including *clin.iobio*. *Clin.iobio* integrates a common set of analysis steps in a typical genomic diagnostic workflow into a single, easy-to-use, and visual web-based application that allows all team members to contribute their expertise to the diagnostic process, without imposing a significant time burden. The flexible design of *clin.iobio* allows for a comprehensive genomic analysis using both phenotype-driven and variant-driven prioritization approaches. 

Within a phenotype-based analysis, *clin.iobio* allows for on-demand updating of phenotype terms and gene lists. This allows team members to dynamically expand or refine phenotype and gene lists. For example, in the case we described here, a user could have been more permissive, and only required genes to be associated with two or more HPO terms. This less stringent filtering would have expanded the gene list to 45 genes. In addition to HPO, users can also utilize the integrated GTR [19] and Phenolyzer [20] resources to generate phenotype-associated gene lists—an approach we published previously [21]. This flexibility allows *clin.iobio* to go beyond a linear workflow, and provides the opportunity for revision, exploration, and reanalysis of previously negative cases. In conclusion, *clin.iobio* provides a comprehensive, web-based genomic analysis platform that enables team-based diagnostic decisions that have the potential to significantly impact clinical practice and patient care. 

## 4. Materials and Methods

### 4.1. System Overview

*Clin.iobio* utilizes and coordinates multiple components and tools within the *iobio* suite of visual web-based genomics tools. These include tools for reviewing data quality metrics (based on *bam.iobio* [22] and *vcf.iobio*), generating lists of genes associated with specific phenotypes and genetic disorders (based on *genepanel.iobio* [21]), and variant prioritization and interpretation (based on *gene.iobio* [12]). Combining these code bases, *clin.iobio* passes the outputs from individual steps to subsequent steps, resulting in a complete start-to-finish diagnostic workflow. Critically, the final step of *clin.iobio* produces a research report, summarizing the case and the findings that were noted during the analysis.

### 4.2. File Input/Output

*Clin.iobio* accepts file-format-compliant PED files, indexed BAM/CRAM files, and indexed (unannotated or annotated) VCF files. These files can be provided via a publicly accessible URL or from a user’s local machine, or from a combination of the two locations. As with all *iobio* apps, *clin.iobio* streams relevant portions of the data and displays the data visually in a web browser; no data are uploaded and no genomic data are stored on *iobio* servers. *Clin.iobio* is a JavaScript application that interfaces with cloud-based *iobio* backend services (https://github.com/iobio/iobio-gru-backend, accessed on 17 September 2021). *Iobio* backend services utilize application programming interface (API) methods to ensure the data and annotations displayed are up-to-date. Furthermore, this architecture delineates application and data processing logic, with the *clin.iobio* front-end displaying visualizations and coordinating secure HTTPS requests to the backend. *Clin.iobio* provides an exportable PDF research report that summarizes the user’s findings. *Clin.iobio* has also been integrated into *Mosaic*—a commercial and collaborative genomic data platform developed by Frameshift Labs (https://frameshift.io/, accessed on 5 January 2022). With this *Mosaic* integration, *clin.iobio* analyses can be saved and relaunched at any time.

### 4.3. Sequencing Data Coverage and Alignment

*Clin.iobio* displays sequencing data coverage visualizations based on the data returned from *iobio* backend services. This coverage-based iobio backend service utilizes SAMtools [23] for region-based queries of CRAM/BAM alignment files, and to determine coverage across a gene or a given region, such as an exon. This coverage information is visualized in both the *Review case* and *Review variants* steps.

### 4.4. IGV Integration

The web-based JavaScript version of the Integrative Genomics Viewer (IGV) [17], called igv.js (https://github.com/igvteam/igv.js/, accessed on 17 September 2021), is integrated into the *Review variants* step (within *gene.iobio*).

### 4.5. Variant Annotation

Variant annotation is performed in the variant review step of *clin.iobio* (using *gene.iobio*), in a region-specific manner, with the data streamed back to *clin.iobio*. This variant annotation service includes tabix [24] (for region-based querying of indexed VCF files), vt [25] (for sample subsetting, variant decomposition, normalization, and transformation), VEP [26] (for transcript-aware annotation of variants with functional consequence, impact, ClinVar [13] significance, REVEL [15] score, HGVS [27], and dbSNP [28] ID), and bcftools (for determining variant population allele frequency in gnomAD) (https://github.com/samtools/bcftools, accessed on 5 January 2022). GnomAD [14] population allele frequencies, as well as heterozygous and homozygous allele counts, are provided. The phlyoP [29] conservation scores and multiple-species sequence alignment visualizations rely on UCSC [30] genome tracks to display multiple-organism sequence alignments surrounding a given variant.

### 4.6. Gene–Disease Association

The GENCODE [31] and RefSeq [32] are used to provide gene name typeahead and autocomplete functionality. The *Select phenotypes* step (based on *genepanel.iobio*) integrates Phenolyzer [20], ClinPhen [33], and the HPO [6], allowing the user to enter a phenotype term and automatically generate a list of genes associated with that phenotype. Up-to-date gene–disease association data from OMIM [34] are retrieved via their web API, while PubMed articles associated with a particular gene are retrieved using the web API NCBI E-utils [35].

### 4.7. External Resources and Databases

Numerous public datasets are utilized to present up-to-date gene and variant annotations to the user. These external resources and databases are kept up-to-date using iobio backend services built around the individual data type. For instance, the ClinVar resource is maintained via a backend service that retrieves the latest ClinVar VCF on a weekly basis. Numerous other external links are provided at the gene- and variant-specific levels, including MARRVEL [36], VarSome [37], OMIM [34], DECIPHER [38], GeneCards [39], GTEx [40], HumanMine [41], PubMed, UniProt, the Human Protein Atlas [42], and the UCSC Browser [30].

### 4.8. Deployment, Usage and Availability

*Clin.iobio* is publicly available and free to use for academic purposes at https://clin.iobio.io/, accessed on 5 January 2022. Commercial use is licensed through Frameshift Labs, Inc., Cambridge, MA, USA (https://frameshift.io/, accessed on 5 January 2022). The University of Utah and the Utah Center for Genetic Discovery maintain an institutional version of *clin.iobio* for use by our clinical teams and genetics researchers at our institute. *Clin.iobio* was developed and optimized for the Chrome browser, with additional support for the Firefox and Safari browsers.

## 5. Conclusions

Genomic testing is increasingly becoming a first-line diagnostic approach for critically ill newborns as well as patients with rare and undiagnosed diseases. In both of these settings, experts from many specific disciplines contribute their unique expertise to a genetic and clinical diagnosis for the patient. *Clin.iobio* is an approachable diagnostic genomic analysis workflow specifically designed to engage all members of the clinical diagnostic team, and serves to accelerate the incorporation of genomic findings into patient care.

## Data Availability

No new data were created in this study. Data sharing is not applicable to this article.
